# Expression of concern: Chemoselective hydration of nitriles to amides using hydrated ionic liquid (IL) tetrabutylammonium hydroxide (TBAH) as a green catalyst

**DOI:** 10.1039/d4ra90115k

**Published:** 2024-09-30

**Authors:** Hojat Veisi, Behrooz Maleki, Mona Hamelian, Samaneh Sedigh Ashrafi

**Affiliations:** a Department of Chemistry, Payame Noor University Tehran 19395-4697 Iran hojatveisi@yahoo.com; b Department of Chemistry, Hakim Sabzevari University Sabzevar 96179-76487 Iran b.maleki@hsu.ac.ir +98-51-4401300 +98-51-44013324

## Abstract

Expression of concern for ‘Chemoselective hydration of nitriles to amides using hydrated ionic liquid (IL) tetrabutylammonium hydroxide (TBAH) as a green catalyst’ by Hojat Veisi *et al.*, *RSC Adv.*, 2015, **5**, 6365–6371, https://doi.org/10.1039/C4RA09864A.

*RSC Advances* is publishing this expression of concern in order to alert readers that concerns have been raised over the integrity of the data published in this article. The authors have been contacted but have not responded to requests to provide raw data. An expression of concern will continue to be associated with the article until a conclusive outcome is reached.

Laura Fisher

16th September 2024

Executive Editor, *RSC Advances*

